# Evolution Characteristics of Surface Water Quality Due to Climate Change and LUCC under Scenario Simulations: A Case Study in the Luanhe River Basin

**DOI:** 10.3390/ijerph15081724

**Published:** 2018-08-11

**Authors:** Wuxia Bi, Baisha Weng, Zhe Yuan, Mao Ye, Cheng Zhang, Yu Zhao, Dengming Yan, Ting Xu

**Affiliations:** 1 State Key Laboratory of Simulation and Regulation of Water Cycle in River Basin, China Institute of Water Resources and Hydropower Research, Beijing 100038, China; biwuxia_1992@163.com (W.B.); yuanzhe_0116@126.com (Z.Y.); yemao@iwhr.com (M.Y.); zhangc@iwhr.com (C.Z.); 18519500795@163.com (D.Y.); xuting900515@163.com (T.X.); 2 College of Hydrology and Water Resources, Hohai University, Nanjing 210098, China; 3 Changjiang River Scientific Research Institute, Wuhan 430010, China; 4 State Key Laboratory on Environmental Aquatic Chemistry, Research Center for Eco-Environmental Sciences, Chinese Academy of Sciences, Beijing 100085, China; yuzhao@rcees.ac.cn; 5 College of Environmental Science and Engineering, Donghua University, Shanghai 201620, China

**Keywords:** surface water quality, climate change, LUCC, model simulation, Luanhe River Basin (LRB)

## Abstract

It is of great significance to study the effects and mechanisms of the key driving forces of surface water quality deterioration—climate change and LUCC (land use and land cover change). The Luanhe River Basin (LRB) in north-eastern China was examined for qualitatively and quantitatively assessing the responses of total nitrogen (TN) and total phosphorus (TP) loads on different climate scenarios and LUCC scenarios. The results show that from 1963 to 2017, the TN and TP loads basically presented a negative correlation with the temperature change (except for winter), while showing a significant positive correlation with the precipitation change. The incidence of TN pollution is sensitive to temperature increase. From 2020 to 2050, the annual average loads of TN and TP were slightly lower than from 1963 to 2017. The contribution of rising temperature was more significant on nutrient loads. Also, the incidence of TN pollution is sensitive to the future climate change. Under LUCC scenarios, the TN and TP loads and pollution incidence increased correspondingly with the decrease of natural land. The evolution characteristics analysis can provide support for the effect and adaptation-strategies study of climate change and LUCC on surface water quality.

## 1. Introduction

With the economic development and population increase, the global shortage of water resources is grim [1] and water pollution is also exacerbated [2]; surface freshwater pollution has become a major public hazard [3]. At present, 40% of the rivers in the world have been polluted to varying degrees, and this statistic shows an upward trend [4]. Most basins in China have also been polluted to different degrees, and the water environment quality has been deteriorating day by day [5,6]. The water pollution presents an increasing trend with extension from tributaries to mainstream, diffusion from region to basin, penetration from surface to underground, and spreading from urban to rural areas [5,7,8]. According to the “2016 State of the Environment”, among the 1940 assessment sections of surface water, water quality in Class I, II, III, IV, V and inferior V accounted for 2.4%, 37.5%, 27.9%, 16.8%, 6.9% and 8.6%, respectively [9]. The detailed information about water quality parameters in different levels can be found in “Environmental Quality Standards for Surface Water’’ [10]. The surface water quality has been improved compared with the previous years, however, the water pollution in the Haihe River Basin (including the Luanhe River) is more serious. Therefore, it is of great significance to study the surface water quality of the Luan River.

At present, the main factor affecting surface water quality is anthropogenic activities [11]. As the point-source pollution has been controlled to some extent, non-point-source pollution becomes a new problem that needs to be solved urgently [5,12]; especially the LUCC (land use and land cover change), which is the main factor affecting non-point-source pollution [13]. Also, the change of climatic conditions in the future may lead to some uncertainties in water environment quality improvement, risk prevention and control effectiveness. In particular, under climate change, the intensity and frequency of extreme climate events such as heavy rainfalls, floods and droughts will increase, which increases the possibility of pollution incidents, and also augments the difficulty of water-environment risk prevention [14,15]. Hence, climate change and LUCC are the key driving forces for surface water quality evolution.

The impact of climate change on the natural environment, living creatures and others cannot be underestimated [16]. There are relatively much more research results of the climate change impact on the amount of water resources, but the response of water quality to climate change needs to be further studied [17]. The drastic changes of temperature and precipitation will result in water pollution [18], which directly or indirectly affects the water quality in the basin. Water temperature changes will affect the water density, surface tension, viscosity and other aspects, and dissolved oxygen content will also change, thus breaking the normal aquatic ecological balance [19,20,21]. Temperature change will also affect the photosynthesis, the chemical reaction rate, the toxicity changes of various pollutants, and the microbial degradation ability of aquatic organisms [22]. Precipitation change will affect the occurrence of droughts and floods, and also will exert a subtle influence on the basin water quality. Precipitation decrease will prompt the exploitation of groundwater, then cause groundwater pollution; precipitation increase will aggravate atmospheric deposition and surface erosion, thus more pollutants will be brought into water [23].

Rapid urbanization changes the land use pattern, which adversely affects the water cycle, and augments the extreme-event possibility, thus leading to a deteriorating water environment [24,25,26]. The change of land use patterns will lead to the change of the underlying surface properties, which will also affect the hydrological cycle [27]; the basin runoff production and concentration will change, thus further affecting the surface water quality in the basin [28]. Studies show that different land uses have different impacts on water quality in the basin [29,30]; for example, woodland and grassland can greatly improve water quality [31], while industrial land, agricultural land and residential land use have a positive correlation with pollutant concentrations [32,33].

For instance, there has been scant study of how climate change and LUCC affect water quality on the basin scale. Combined with the lack of long-term water quality measured data, models are mainly used to simulate and predict the impact of climate change and LUCC on water quality [34].

In the past 50 years, both the annual precipitation and natural runoff in the Luanhe River Basin (LRB) had a decreasing trend. However, the demand for water resources has been increasing with the socioeconomic development. Therefore, the drought situation in the river basin has become increasingly severe, and also the water quality and hydrology conditions continue to deteriorate. According to the statistical analysis of water quality in the recent 10 years, the polluted rivers (Class IV and below) accounted for 43.2% of all evaluated rivers [35]. The monitoring results showed that the main pollution indicators of surface water in the LRB were ammonia nitrogen, total nitrogen (TN) and total phosphorus (TP) [36]. The dissolved oxygen (DO) and ammonia nitrogen in the upper reaches of the Luanhe River changed obviously; the comprehensive pollution index of water quality in the midstream fluctuated basically between 0.2 and 0.4, and presented a downward trend in the downstream [37]. As nitrogen and phosphorus are the main pollutants contributing to the deterioration of water quality in the Luanhe River, they were chosen as influence factors of water quality for analysis.

**Figure 1:**
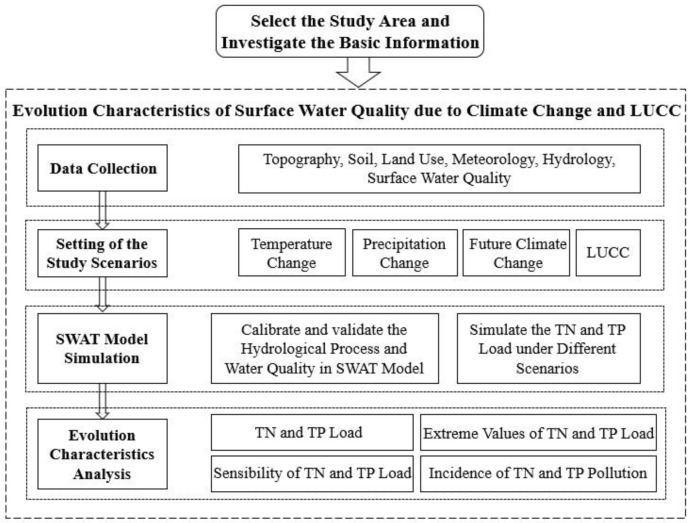
The overview of the study. LUCC refers to land use and land cover change; SWAT refers to Soil and Water Assessment Tool; TN means total nitrogen (TN); TP means total phosphorus.

The main objectives of our study were to: (i) analyze the load change of TN and TP flowing into the Luanhe River under the influence of temperature and precipitation change; (ii) predict the pollution incidence of TN and TP under future climate change; and (iii) investigate the evolution characteristics of TN and TP under land use change along the Luanhe River. Under four different scenario simulations (3 of climate change, 1 of LUCC), the evolution characteristics of total nitrogen (TN) and total phosphorus (TP) load were investigated, which were based on the calibration and validation of hydrological process and water quality of the SWAT (Soil and Water Assessment Tool) model. To explore the evolution characteristics in each scenario, the main evaluation index contains four parts: TN and TP load, extreme values of TN and TP load, sensibility of TN and TP load, incidence of TN and TP pollution (Figure 1). The broad implication of the present research is to better understand the historical evolution characteristics of surface water quality under climate change and LUCC, and also to predict the future evolution trends. The impact analysis of surface water quality under climate change can guide the adaptation strategies to the changed environment. The evolution mechanism of surface water quality under LUCC would provide references for the future planning of agricultural development, soil and water conservation, and so on.

## 2. Materials and Methods

### 2.1. Study Site

The LRB (39°10′–42°30′ N, 115°30′–119°15′ E), located at the northern Haihe River Basin, is one of the four major rivers in the Haihe River Basin. The Luanhe River originates from the foot of the Bayandun-Tuergu Mountain and discharges into Bohai Bay, Hebei Province [38,39]. The river flows through 27 cities and counties in Hebei Province, Inner Mongolia Autonomous Region and Liaoning Province, with watershed area of 44,750 km^2^. The basin has a population of 5.4442 million with population density of 122 persons/km².

The elevation of the LRB decreases from north to south, with three types of landforms: plateau, mountain and plain. The plateau is located at the northern part of the basin, with an altitude of 1400 to 1600 m. The mountainous landform is distributed from the south of the plateau to the north of the plain. The slope is generally between 20° and 40°. The plain is in the southern part of the basin, with a longitudinal slope of 1/300 to 1/1000.

The region experiences humid, semihumid and semiarid temperate continental monsoon climate from southeast to northwest. It is characterized by four distinct seasons, significant monsoon, concentrated precipitation, rain and heat over the same period, diverse terrain, complex climate, and other characteristics. The interannual precipitation variation is large, and the mean annual precipitation in the basin varies between 390 and 800 mm. The seasonal distribution of precipitation is significantly heterogeneous, with differences in each month, especially concentrated in summer with a volume of 260 to 560 mm, accounting for 67% to 76% of the annual precipitation. The multiyear average water surface evaporation in the basin is about 950 to 1150 mm.

### 2.2. Data Sources

This study used the SWAT model to simulate the TN and TP load in the basin. The input data of the SWAT model contains six major types, that is, topography, soil, land use, meteorology, hydrology and surface water quality (Table 1). Future meteorological data is based on the simulation results of the future climate prediction scenario model. The surface water quality data from 2015 to 2017 was based on monthly measurements in the downstream water quality monitoring site (Luanxian Station). The samples were analyzed on the basis of the Environmental Quality Standards for Surface Water (GB3838-2002) [10].

Future climate prediction is based on the scenarios considering the emissions of greenhouse gases and aerosols. Representative Concentration Pathways (RCPs) is a new scenario developed in the IPCC’s (Intergovernmental Panel on Climate Change’s) Fifth Assessment Report, and it was used as climate scenarios, including RCP2.6, RCP4.5, RCP6.0 and RCP8.5 [40,41]. The selected climate scenario model was based on the interpolated, revised results of five sets of global climate scenarios (GFDL-ESM2M, HADGEM2-ES, IPSL-CM5A-LR, MIROC-ESM-CHEM and NORESM1-M) [42,43] provided by the Inter-Sectoral Impact Model Intercomparison Project (ISI-MIP).

According to the existing research, three climate scenarios—RCP2.6, RCP4.5 and RCP8.5—were selected as future climate prediction scenario models to analyze future climate change in the LRB to 2050. The evaluation and optimization of climate prediction scenario models has been described in earlier studies [44,45].

### 2.3. Study Scenarios

It is difficult to assess the effect of climate change on water quality by separating the anthropogenic activities’ impacts. Hence, we set up climate change scenarios to analyze the impact of climate change. To assess the influence of climate change and LUCC on the surface water quality in the LRB, four scenarios were designed, as follows (Table 2).

Temperature Change scenario. The variation laws of TN and TP in the basin under single temperature change were investigated on the basis of the historical meteorological data from 1963 to 2017. The single temperature change means that the temperature was set increasing/decreasing by 1 °C and 2 °C, and the other meteorological factors were kept unchanged. The land use datasets of 1985, 2000 and 2014 were used to verify the universality of the variation laws.

Precipitation Change scenario. Similar to the Temperature Change scenario setting, the variation laws of TN and TP in the basin under single precipitation change were analyzed. The single precipitation change refers to the precipitation increasing/decreasing by 10% and 20% only.

Future Climate Change scenario. To predict the evolution of TN and TP in the basin under future climate change compared with historical data, three future climate prediction scenario models RCP2.6, RCP4.5 and RCP8.5 were selected to simulate the climate change from 2020 and 2050 in the LRB, combined with the land use data of 2014.

LUCC scenario. The evolution differences of TN and TP in the basin with the land use datasets of 1985, 2000 and 2014 were compared to reveal the impact of the underlying surface change. Various types of land use were broadly divided into three categories, that is, natural land use, human activities land use and undeveloped land use. The proportion changes of each category in 1985, 2000 and 2014 were identified (Table 3). This analysis applied different land use datasets, while the other factors that can influence TN and TP loads, such as WWTP (wastewater treatment plants) efficiencies, “good practice” in agriculture, and so on, were kept unchanged.

The above scenario analyses were based on model simulation and prediction. The key study preconditions are the distributed water quantity and water quality coupling model, and the long-term series of temperature and precipitation data.

### 2.4. SWAT Model

The SWAT model, as a distributed hydrological model, has been widely used to assess the long-term impacts of different climatic conditions and land cover changes on sediment, nutrients and so on [46]. The transformation of different databases [44,47,48,49] will not be explained in detail in this manuscript.

ArcSWAT2012 (a public domain model jointly developed by USDA Agricultural Research Service and Texas A&M AgriLife Research) was used to simulate four designed scenarios. In this study, the LRB was divided into 88 sub-basins with the smallest catchment area threshold defined as 250 km^2^. The model construction, calibration and validation details of the hydrological process can be found in references [44,50].

To explore the characteristics of TN and TP under different scenarios, besides the hydrological calibration and validation, the calibration and validation of water quality is needed as well. As there has been a lack of long-term continuous monitoring data of surface water quality in the Luanhe River, the calibration and validation with the observed water quality dataset from 2015 to 2017 was conducted. In the SWAT model, nitrogen and phosphorus were divided into different forms for cyclic conversion when simulating the TN and TP in the river. Equations (1) and (2) calculate TN and TP load, respectively.

(1) $$\mathrm{TN}=\mathrm{ORGN}+{\mathrm{NO}}_{3}+{\mathrm{NH}}_{4}+{\mathrm{NO}}_{2},$$

where TN is the total nitrogen load, ORGN is the organic nitrogen load, ${\mathrm{NO}}_{3}$ is the nitrate nitrogen load, ${\mathrm{NH}}_{4}$ is the ammonia nitrogen load, and ${\mathrm{NO}}_{2}$ is the nitrite nitrogen load.

(2) TP=ORGP+MINP,

where TP is the total phosphorus load, ORGP is the organic phosphorus load, and MINP is the mineral phosphorus load.

### 2.5. Data Analysis

Based on the output results in SWAT, the average monthly load and average annual load of TN and TP under different scenarios were analyzed. To better understand the monthly variation trends of TN and TP load, the extreme values were further screened. The annual mean, three-times annual mean and five-times annual mean of TN and TP load from 1963 to 2017 were chosen as three benchmarks. The percentages above (including equal to) the three benchmarks in different scenarios were calculated. Meanwhile, the sensibility of the water quality index in different scenarios was considered.

To evaluate the water quality situation, the incidence of water pollution was further explored. A single-factor evaluation method was chosen as the water quality evaluation method. The indicators for the assessment are TN and TP. For the unified analysis, the surface water is considered contaminated with TN and TP concentration greater than Class IV in the Environmental Quality Standards for Surface Water (GB3838-2002). The thresholds of TN and TP are 1.5 mg/L and 0.3 mg/L, respectively. Equation (3) calculates the incidence of water pollution.

(3) $$p=\frac{m_{p}}{M},$$

where p is the incidence of water pollution, $m_{p}$ is the number of polluted months during the study period, M is the total number of months.

## 3. Results

### 3.1. Model Calibration and Validation

**Figure 2:**
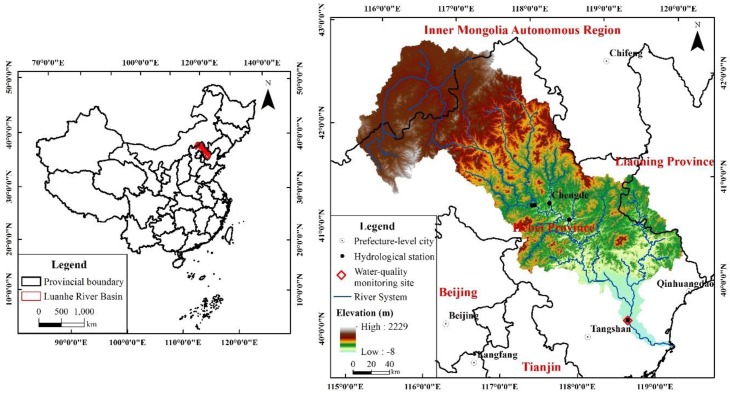
The Luanhe River Basin (LRB).

**Figure 3:**
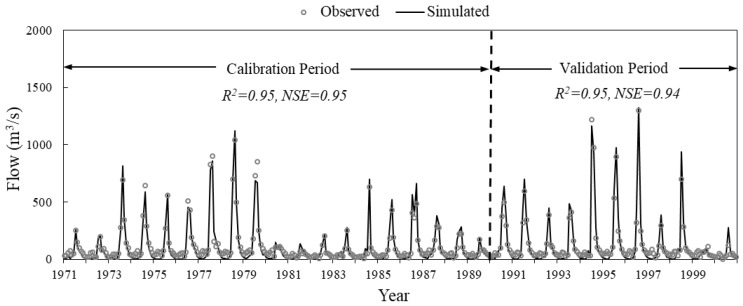
The hydrological calibration and validation results of the Luanxian Station.

**Figure 4:**
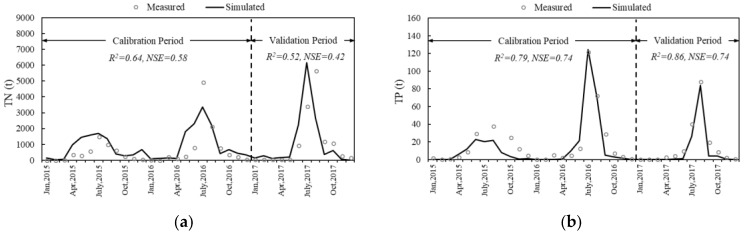
The calibration and validation results of TN load (**a**) and TP load (**b**) in the SWAT model.

For the hydrological calibration and validation process in the SWAT model [43,49], flow data of five hydrological stations (Figure 2) was applied. The average linear regression correlation coefficient (R^2^) and Nash–Sutcliffe efficiency coefficient (NSE) of the five stations were 0.85 and 0.83, respectively, during the calibration period. The average R^2^ and NSE were 0.85 and 0.74, respectively, during the validation period (Table 4). To limit the article length, only the hydrological calibration and validation results of the Luanxian Station are plotted in this paper (Figure 3), since the Luanxian Station is the monitoring station for both hydrology and water quality (Figure 2). The R^2^ and NSE were 0.95 and 0.95, respectively, during the calibration period, and 0.95 and 0.94, respectively, during the validation period (Figure 3). To calibrate the water quality indicators (TN and TP), the measured water quality data from 2015 to 2017 were applied for the calibration and validation. Figure 4 presents the water quality calibration and validation results. For TN, the R^2^ and NSE were 0.64 and 0.58, respectively, during the calibration period, and 0.52 and 0.42, respectively, during the validation period. For TP, the R^2^ and NSE were 0.79 and 0.74, respectively, during the calibration period, and 0.86 and 0.74, respectively, during the validation period (Table 4).

As for the future climate prediction scenario model, the prediction results showed that the annual precipitation presented an increasing trend in the LRB from 2020 to 2050, with significant alternation of wet and dry. The precipitation changes were basically the same in the RCP2.6 and RCP4.5. Significant increase presented in the downstream plain area (more than 10% in both RCP2.6 and RCP4.5) and in the upper reaches (more than 10% in RCP2.6 and more than 5% in RCP4.5), while in the RCP8.5, the areas with large precipitation increases were located in the source of the river, and the upper and middle reaches (more than 10%). The mean annual temperature will keep increasing in the LRB. The annual temperature change was in accordance with the historical period, while the monthly temperature had remarkable increases in late spring, early autumn and winter. The average annual temperature would increase above 1.75 °C in 2050 compared with the reference period in the whole LRB, with much more rapid increase in the upper plateau and the lower plain. However, the average annual temperature in the RCP8.5 was significantly higher than that in RCP2.6 and RCP4.5.

### 3.2. Temperature Change Scenario

**Figure 5:**
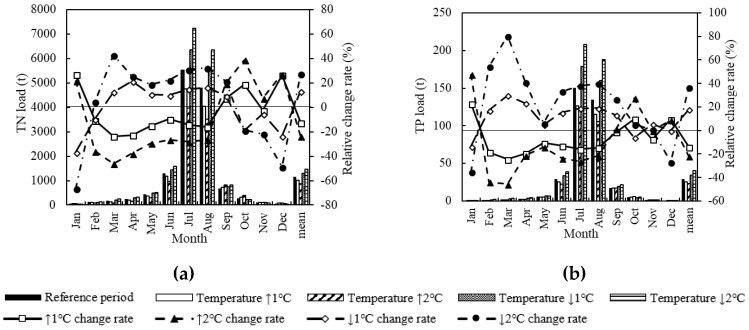
Monthly change of TN load (**a**) and TP load (**b**) under the Temperature Change scenario.

In the Temperature Change scenario, the TN and TP load basically showed a negative correlation with temperature change except for winter (from October to January) (Figure 5). Under the land use dataset of 2014, when temperature increased 1 °C, 2 °C, −1 °C or −2 °C compared with the reference period (from 1963 to 2017), the monthly TN load increased by −23.6% to 27.3%, −46.0% to 38.9%, −36.6% to 21.5%, and −66.5% to 42.8%, respectively; the monthly TP load increased by −24.5% to 22.9%, −45.5% to 47.7%, −13.5% to 29.8%, and −35.3% to 80.6%, respectively; the mean annual TN load increased by −12.4%, −23.6%, 13.3% and 27.4%, respectively; and the average annual TP increased by −14.0%, −21.8%, 17.9% and 36.9%, respectively.

The percentage above (including equal to) the three benchmarks was calculated (Table 5). Under the land use dataset of 2014, the extreme value proportions of TN load above the three benchmarks were 19.6% to 22.6%, 7.5% to 11.8%, and 3.9% to 8.0%, respectively; the extreme value proportion of TP loads were 17.3% to 21.6%, 8.0% to 11.6%, and 4.6% to 9.0%, respectively. As the temperature increases (decreases), the proportions of the three types of extreme values decrease (increase). The greater the absolute change value of the temperature, the larger the proportion change of the extreme value.

**Figure 6:**
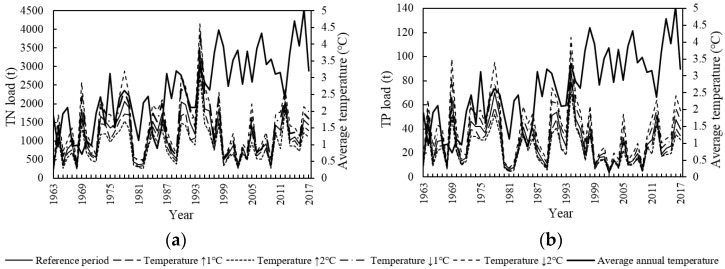
TN load (**a**), TP load (**b**) and average annual temperature variations under the Temperature Change scenario.

Figure 6 plots the sensitivity of TN/TP load to average annual temperature under the Temperature Change scenario. The load altered up and down as the average annual temperature increased. The load changed sharply within the temperature range of 2.6 to 3.0 °C, which was the sensitive temperature range of water quality change.

Under the Temperature Change scenario, the incidence of TN and TP pollution shows different variation trends (Table 6). The incidence of TN pollution shows a positive correlation with temperature change. The greater the absolute change value of the temperature, the larger the change of the incidence. However, there is basically a negative correlation between temperature change and variation of incidence of TP pollution. The TN pollution is more serious than TP pollution in the LRB.

### 3.3. Precipitation Change Scenario

**Figure 7:**
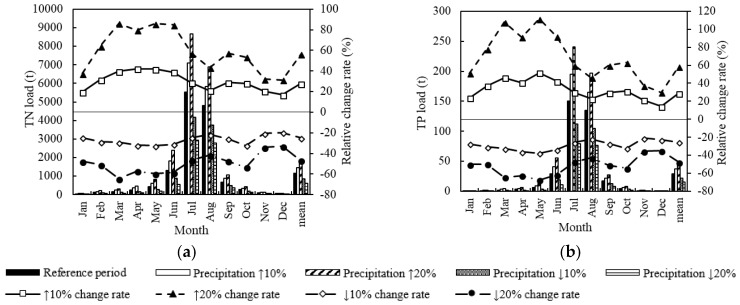
Monthly TN load change (**a**) and TP load change (**b**) under the Precipitation Change scenario.

In the Precipitation Change scenario, the TN and TP load presented a significantly positive correlation with the precipitation change (Figure 7). Under the land use of 2014, when precipitation increased by 10%, 20%, −10% and −20% compared with the reference period, the monthly TN increased by 17.0% to 42.3%, 32.0% to 86.2%, −32.3% to −19.7% and −64.6% to −33.1%, respectively; the monthly TP increased by 14.4% to 51.6%, 29.8% to 111.6%, −37.6% to −20.8% and −67.5% to −34.9%, respectively; the average annual TN increased by 27.7%, 56.3%, −24.9% and −47.0%, respectively; and the average annual TP increased by 28.8%, 58.7%, −25.4% and −47.6%, respectively.

As can been seen in Table 7, under the land use of 2014, the extreme value proportions of TN load above the three benchmarks were 12.4% to 27.5%, 5.6% to 15.0%, and 2.3% to 10.1%, respectively; the extreme value proportions of TP load were 11.8% to 24.0%, 5.9% to 14.4%, and 2.8% to 9.8%, respectively. As the precipitation increases, the proportions of the three types of extreme values increase, and vice versa. The greater the absolute change value of the precipitation, the larger the proportion change of the extreme value.

**Figure 8:**
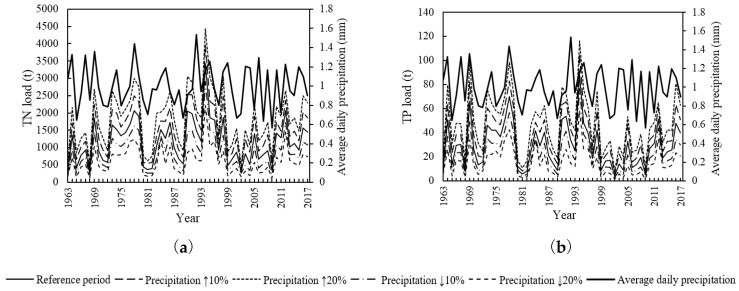
TN load (**a**), TP load (**b**) and average daily precipitation variations under the Precipitation Change scenario.

The correlation between TN/TP load and the average daily precipitation was basically proportional. The load varied significantly within the precipitation range of 0.9 to 1.0 mm, which was the sensitive precipitation range of water quality change (Figure 8).

TN and TP pollution showed no obvious variation trend under different Precipitation Change scenarios (Table 8). Among most Precipitation Change scenarios, the incidence of TN and TP pollution will be reduced slightly compared with the reference period. When precipitation increases by 20%, the incidence of TN and TP pollution decreases the most. Also, the TN pollution is more serious than TP pollution in the LRB.

### 3.4. Future Climate Change Scenario

**Figure 9:**
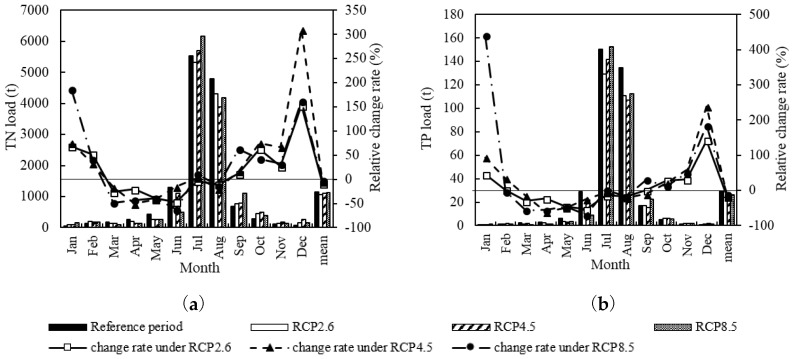
Monthly TN load change (**a**) and TP load change (**b**) under the Future Climate Change scenario.

The monthly load of TN and TP simulated by RCP2.6, RCP4.5 and RCP8.5 varied differently in the future period. However, the variation trends of TN and TP were roughly the same in each scenario model (Figure 9). From the perspective of monthly mean, the TN and TP load in three future climate scenario models generally showed decreasing trends from March to August and increasing trends from September to February. The monthly load in RCP2.6 was relatively higher in August. The monthly load in RCP4.5 was significantly higher in late spring, early summer (June), autumn and winter. The monthly load in RCP8.5 was more remarkable in summer, while for annual mean, the TN and TP load was slightly lower than that in the reference period. The load increased according to the following order: RCP 2.6 < RCP 4.5 < RCP 8.5.

Table 9 lists the analysis results of extreme values. The proportion of extreme values in RCP2.6, RCP4.5 and RCP8.5 was slightly lower than that in the reference period. The variations of extreme proportion presented a non-unified law. The proportion of extreme values based on three benchmarks—annual mean, three-times annual mean and five-times annual mean—presented in the following orders: RCP4.5 > RCP2.6 > RCP8.5, RCP8.5 > RCP4.5 > RCP2.6, and RCP2.6 > RCP8.5 > RCP4.5, respectively.

Table 10 presents the incidence of TN and TP pollution under the Future Climate Change scenario. The incidence of TN pollution increased in three different RCPs, with increasing order of reference period < RCP8.5 < RCP2.6 < RCP4.5. The incidence of TN pollution augmented about 6% to 8% in three RCPs compared with the reference period. Meanwhile, the incidence of TP pollution decreased. In RCP2.6 and RCP8.5, there is no pollution possibility in the future. Obviously, the TN pollution is more serious than TP pollution.

### 3.5. LUCC Scenario

**Figure 10:**
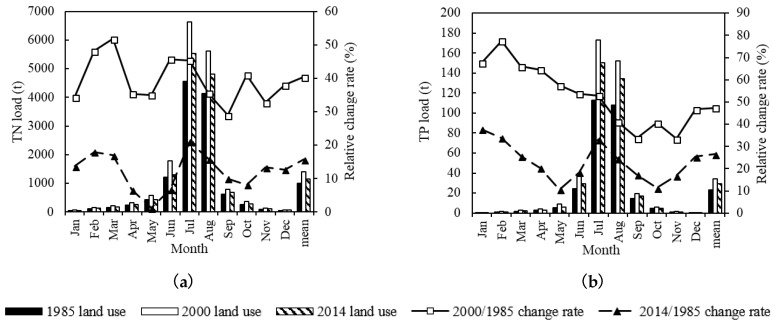
Monthly TN load change (**a**) and TP load change (**b**) under the LUCC scenario.

With non-change of climatic factors, the TN and TP load in the reference period (from 1963 to 2017) changed differently under the land use of 1985, 2000 and 2014 (Figure 10). Compared with land use of 1985, the monthly TN and TP load increased under the land use of 2000 and 2014, with larger increase in 2000 than in 2014. The monthly TN load in land use of 2000 and 2014 increased by 29.0% to 51.8%, and 1.2% to 21.1%, respectively. The monthly TP load increased by 33.4% to 77.5%, and 10.6% to 37.8%, respectively. From the perspective of annual average load, the TN load increased by 40.4% and 15.7%, respectively, and the TP load increased by 47.5% and 26.6%, respectively.

The incidence of TN and TP pollution under different land use scenarios increases with the following order: land use of 2000 > land use of 2014 > land use of 1985 (Table 11). This trend is the same with TN and TP load variation under different land uses. Meanwhile, the TN pollution is more serious than TP pollution.

## 4. Discussion

### 4.1. Effect of Single Climatic Factor Change on Surface Water Quality

In the Temperature Change scenario, the TN and TP loads (water pollution extreme events) were basically inversely proportional to the temperature change (excluding winter). When the temperature increased (decreased) 1 °C, the load decreased (increased) by 12% to 19%, and the occurrence probability of extreme events decreased (increased) by 1% to 2%. The trend can be interpreted as follows. When the temperature increases, the evaporation increases, the basin runoff decreases and the pollutant degradation coefficient of the water body increases [22], thus the pollution into the river decreases. In addition, the load of TN and TP in the LRB was remarkably high from June to September but lower in winter. This trend is the combined effects of growing season, leaf litter, water temperature and other contributing factors. In summer, the synthesis rate of water pollutants is greater than the decomposition rate; the scouring effect is obvious with large runoff. In winter, farming areas reduce as the runoff and the temperature are relatively low. The incidence of TN pollution shows a positive correlation with temperature change, while there is no obvious correlation between temperature change and variation of incidence of TP pollution. It can be inferred that the TN production speed is faster than its composition speed when temperature increases, while no remarkable rule is found with TP.

In the Precipitation Change scenario, the TN and TP loads (water pollution extreme events) were significantly proportional to the precipitation change. When the precipitation increased (decreased) by 10%, the load increased (decreased) by 23% to 30%, and the occurrence probability of extreme events increased (decreased) by 3% to 4%. The reasonable explanation is that when the precipitation increases, the basin runoff increases and the pollutant degradation coefficient of the water body increases [22], thus the pollution into the river decreases. There is no obvious variation trend of TN and TP pollution under different Precipitation Change scenarios. Maybe the incidence of TN and TP pollution is not sensitive to the precipitation change.

In terms of climate change, under single climatic factor (temperature/precipitation) change scenarios, the variation trend of TN and TP loads in the LRB are similar to other studies [51]. It has been verified that evolution laws of TN and TP load were similar with different land use datasets of 1985, 2000 and 2014.

### 4.2. Effect of Future Climate Change on Surface Water Quality

According to the IPCC report, the global average surface temperature in the mid-21st century will change by 1 to 2 °C compared with the historical period from 1986 to 2005, thus the simulation results of the Future Climate Change scenario were reasonable. Much research has been done on water quantity changes in the future [52], this study further analyzed the water quality. The simulation results of the LRB show that from 2020 to 2050, the annual mean of TN and TP load presented a trend of RCP2.6 < RCP4.5 < RCP8.5 < reference period, and the occurrence probability of extreme water pollution events showed a trend of RCP4.5 > reference period > RCP2.6 > RCP8.5. The precipitation-increasing patterns in the three scenarios were similar, but the temperature increased more in spring and summer. As the pollutant decomposition accelerated with temperature rising, the TN and TP load in spring and summer was lower than that in the reference period. The large base load in summer has a significant impact on the mean load, that is why the average annual loads in the three future climate scenario models were slightly lower than that in the reference period. It can be learned that the future temperature-increasing effect is more obvious than the future precipitation-increasing effect on the TN and TP load decreasing. The incidence of TN pollution will increase under all three RCPs, while the incidence of TP pollution will decrease. It can be concluded that the TN load is more sensitive to the future climate change than the TP load.

### 4.3. Effect of LUCC on Surface Water Quality

Compared with land use of 1985, under the land use in 2000 and 2014, the natural land use decreased by 2.88% and 2.46%, respectively; the human activities land use increased by 1.59% and 2.07%, respectively; and the undeveloped land use increased by 1.29% and 0.38%, respectively. The analysis results in the LUCC scenario demonstrate that the TN and TP load increased correspondingly with the decrease of natural land use, the increase of human activities land use and undeveloped land use. The impact of different types of land use on water quality in the LRB shows generally the same trend with other basins [31]. The effect of natural land use decrease on TN and TP load increase was significantly obvious. The more intense human activities, the more damage to natural land, thus the occurrence possibility of water pollution extreme events becomes greater. The data show that the underlying surface change has a significant impact on TN and TP load, as well as the incidence of TN and TP pollution.

## 5. Conclusions

The impact of climate change and LUCC on surface water quality in the LRB was investigated under four designed simulation scenarios: the Temperature Change scenario, Precipitation Change scenario, Future Climate Change scenario and LUCC scenario.

The major conclusions are as follows:

(1) In the past 55 years (from 1963 to 2017), the average annual temperature increased 0.69 °C/10 years. The average daily precipitation varied alternately between 0.5 and 1.6 mm. In the next 30 years (from 2020 to 2050), the average annual temperature will increase by above 0.58 °C/10 years compared with the reference period (from 1963 to 2017). The increase rate of precipitation will be different in the middle and lower reaches of the basin (5% to 10%). The climate in the LRB has been getting warmer and more humid. From 1985 to 2014, the natural land use decreased, the human activities land use increased, while the change rate slowed down.
(2) The TN and TP load was basically inversely proportional to temperature change (except for winter), while it was significantly proportional to precipitation change. Especially in summer, the TN and TP load increased more obviously as precipitation was much higher. Based on the extreme value proportion statistics, the more the temperature or the precipitation changes, the greater the occurrence probability of extreme water pollution events. It can be found that the incidence of TN pollution is sensitive to temperature increase with positive correlation, while it shows a basically negative correlation with TP. Meanwhile, the incidence of TN and TP pollution has no obvious relation with precipitation change.
(3) Through the Future Climate Change scenario simulation, it can be learned that the mean annual TN and TP loads from 2020 to 2050 is likely slightly lower than those in the reference period (from 1963 to 2017). The load might increase according to the following order: RCP2.6 < RCP4.5 < RCP8.5 < reference period. From the point of view of climate change, the temperature rising effect on the TN and TP loads seems to be more obvious than the precipitation increase effect. Additionally, the incidence of TN pollution is potentially sensitive to the future climate change.
(4) The TN and TP loads were different under the land use of 1985, 2000 and 2014. The loads and the incidence of water pollution generally show a trend of 1985 < 2014 < 2000, which is in contrast with the natural land use change (2000 < 2014 < 1985). When the natural land use decreases and the human activities land use increases, the TN and TP loads of the basin will increase as well, and the surface water quality of the basin will deteriorate.

In general, single climatic factor (temperature/precipitation) change will have an obvious effect on the TN and TP loads in the LRB. The underlying surface change will also affect the surface water quality in the basin. The greater the reduction of natural land use, the greater the increase of pollution load in the basin. The incidence of TN and TP pollution is more sensitive to LUCC than single climatic factor change. The incidence of TN pollution will augment in the future, while it will decrease with TP pollution. The evolution characteristics of surface water quality in this paper can provide references for climate change and LUCC change study, and also can support the effect and adaptation-strategies study. Certainly, there are various uncertainties of the impact of climate change and underlying surface change on surface water quality, and following research should focus on basin water quality models, climate models, extreme events, and LUCC causes. TN and TP loads are influenced not only by LUCC, but also by point-source pollution, WWTP efficiencies, “good practice” in agriculture, and so on. The further study can focus on the comprehensive effects of mentioned factors on the TN and TP loads.

## Figures and Tables

**Table 1:** Input data types and main sources.

Data Type	Data Name	Data Source
Topography	Digital Elevation Model (DEM)	-SRTM data (http://srtm.csi.cgiar.org/index.asp)
Soil	China soil database	http://www.soil.csdb.cn
	1:1,000,000 (grid format)	The Second National Land Survey
Land use	Dataset of 1985, 2000 and 2014	Resource and Environment Data Cloud Platform of the Chinese Academy of Sciences (http://www.resdc.cn)
Meteorology	Daily datasets of basic meteorological elements for China’s national surface meteorological stations (V3.0); RCPs (for future simulation)	China Meteorological Data Service Center (http://data.cma.cn)
Hydrology	Monthly observed flow data (five stations, from 1970 to 2000)	Hydrological Yearbook
Surface water quality	Monthly TN and TP load (Luanxian Station, from 2015 to 2017)	Measured data

SRTM represents the Shuttle Radar Topography Mission; RCPs represents the Representative Concentration Pathways.

**Table 2:** Study scenarios.

Study Scenarios		Scenario Settings	Meteorological Data	Land Use Data
Climate Change	Temperature Change	Temperature ± ½ °C	1963–2017	1985, 2000, 2014
	Precipitation Change	Precipitation ± 10%/20%	1963–2017	1985, 2000, 2014
	Future Climate Change	RCP2.6, RCP4.5, RCP8.5	2020–2050	2014
LUCC		Land use dataset of 1985, 2000 and 2014	1963–2017	1985, 2000, 2014

**Table 3:** Different land use proportions under LUCC scenario (%).

Categories	Land Use	1985	2000	2014
Natural land use	Water	1.27	1.73	1.70
	Woodland	45.88	37.67	37.80
	Grassland	26.61	31.49	31.81
	Subtotal	73.76	70.88	71.30
Human activities land use	Arable land	22.50	24.12	22.79
	Residential land	1.42	1.39	3.20
	Subtotal	23.92	25.51	25.99
Undeveloped land use	Wetlands	1.12	1.60	1.76
	Gravel	1.20	2.01	0.95
	Subtotal	2.32	3.61	2.70

**Table 4:** The evaluation of calibration and validation results for hydrological processes and surface water quality.

Indicator	Period	Year	R^2^	NSE
Hydrological process	Calibration	1970–1990	0.85	0.83
	Validation	1991–2000	0.85	0.74
TN	Calibration	2015–2016	0.64	0.58
	Validation	2017	0.52	0.42
TP	Calibration	2015–2016	0.79	0.74
	Validation	2017	0.86	0.74

R^2^ represents the average linear regression correlation coefficient; NSE represents the Nash–Sutcliffe efficiency coefficient.

**Table 5:** The extreme values of TN and TP load under the Temperature Change scenario.

	**TN Extreme Value (%)**				
**Benchmark (t)**	**Reference Period**	**Temperature +2 °C**	**Temperature +1 °C**	**Temperature −1 °C**	**Temperature −2 °C**
1149.74	20.75	19.61	19.77	21.24	22.55
3449.23	9.80	7.52	8.17	10.78	11.76
5748.72	6.21	3.92	4.58	6.86	8.01
	**TP Extreme Value (%)**				
**Benchmark (t)**	**Reference Period**	**Temperature + 2 °C**	**Temperature + 1 °C**	**Temperature −1 °C**	**Temperature −2 °C**
292.43	18.63	17.32	17.81	19.93	21.57
877.30	9.64	8.01	8.66	10.46	11.60
1462.17	6.70	4.58	5.56	7.68	8.99

**Table 6:** The incidence of water pollution (%) under the Temperature Change scenario.

Indicator	Reference Period	Temperature +2 °C	Temperature +1 °C	Temperature −1 °C	Temperature −2 °C
TN	64.24	68.94	66.06	60.61	59.09
TP	1.06	0.61	0.61	0.91	1.97

**Table 7:** The extreme values of TN and TP load under the Precipitation Change scenario.

	**TN Extreme Value (%)**				
**Benchmark (t)**	**Reference Period**	**Precipitation +20%**	**Precipitation +10%**	**Precipitation −10%**	**Precipitation −20%**
1149.74	20.75	27.45	24.18	16.34	12.42
3449.23	9.80	15.03	13.07	6.86	5.56
5748.72	6.21	10.13	7.35	4.41	2.29
	**TP Extreme Value (%)**				
**Benchmark (t)**	**Reference Period**	**Precipitation +20%**	**Precipitation +10%**	**Precipitation −10%**	**Precipitation −20%**
29.24	18.63	24.02	21.73	15.20	11.76
87.73	9.64	14.38	11.11	8.01	5.88
146.22	6.70	9.80	8.66	4.90	2.78

**Table 8:** The incidence of water pollution (%) under the Precipitation Change scenario.

Indicator	Reference Period	Precipitation +20%	Precipitation +10%	Precipitation −10%	Precipitation −20%
TN	64.24	63.03	64.09	64.39	63.33
TP	1.06	0.76	0.91	0.91	0.91

**Table 9:** The extreme values of TN and TP load under the Future Climate Change scenario.

	**TN Extreme Value (%)**			
**Benchmark (t)**	**Reference Period**	**RCP2.6**	**RCP4.5**	**RCP8.5**
1149.74	20.75	18.82	21.51	18.82
3449.23	9.80	8.06	8.60	8.87
5748.72	6.21	5.11	3.49	4.84
	**TP Extreme Value (%)**			
**Benchmark (t)**	**Reference Period**	**RCP2.6**	**RCP4.5**	**RCP8.5**
29.24	18.63	16.13	18.55	15.86
87.73	9.64	7.26	7.80	8.33
146.22	6.70	4.84	3.49	4.57

**Table 10:** The incidence of water pollution (%) under the Future Climate Change scenario.

Indicator	Reference Period	RCP2.6	RCP4.5	RCP8.5
TN	64.24	72.04	72.31	70.16
TP	1.06	0.00	0.81	0.00

**Table 11:** The incidence of water pollution (%) under the LUCC scenario.

Indicator	Land Use of 1985	Land Use of 2000	Land Use of 2014
TN	62.88	67.27	64.24
TP	0.76	2.12	1.06
